# The JAK1 Inhibitor Upadacitinib Curbs Acute Liver Failure via Suppressing IFN-γ/JAK1/STAT1 and TNF-α/NF-κB/MAPK Pathways and Modulating Bax/Bcl-2 Ratio

**DOI:** 10.3390/jox16040140

**Published:** 2026-07-29

**Authors:** Abdulaziz F. Alhussaini, Sara H. Hazem, Eman A. Saad, Mahmoud Elshal

**Affiliations:** 1 Department of Pharmacology and Toxicology, Faculty of Pharmacy, Mansoura University, Mansoura 35516, Egypt; alhussainiph@std.mans.edu.eg (A.F.A.); or melshal@gc.edu.sa (M.E.); 2 Department of Anatomic Pathology, Faculty of Medicine, Mansoura University, Mansoura 35516, Egypt; eman.ashraf.saad@gmail.com; 3 AlGhad College for Applied Medical Sciences, Najran 66243, Saudi Arabia

**Keywords:** upadacitinib, fulminant hepatic failure, LPS/D-galactosamine, hepatoprotection, JAK1/STAT1, MAPK

## Abstract

Acute liver failure (ALF) is a fulminant hepatic syndrome characterized by rapid hepatocellular destruction, severe impairment of liver function, and high mortality. Effective pharmacological interventions capable of limiting early hepatic injury remain lacking. Upadacitinib (UPA), a selective inhibitor for Janus kinase 1 (JAK1) with established anti-inflammatory activity, has not previously been investigated in experimental ALF. Consequently, the current study examined the hepatoprotective potential and underlying mechanisms of UPA in a lipopolysaccharide (LPS)/D-galactosamine (D-GalN)-induced ALF murine model. Mice were pretreated with UPA (10 or 20 mg/kg) prior to LPS/D-GalN challenge. UPA significantly attenuated liver injury, as demonstrated by marked reductions in serum ALT, AST, ALP, and γ-GT levels, together with substantial improvement in hepatic histopathology, attenuation of necroinflammation, and reduction in neutrophil accumulation. UPA also restored hepatic redox balance through reduction in lipid peroxidation and nitrosative stress, alongside enhancement of antioxidant capacity. Mechanistically, UPA suppressed IFN-γ/JAK1/STAT1 signaling and downregulated NF-κB p65 and inducible nitric oxide synthase (iNOS) expression, with subsequent reduction in hepatic TNF-α production. In parallel, UPA inhibited MAPK pathway activation, including ERK1/2, JNK, and p38 signaling. Moreover, UPA attenuated hepatocellular apoptosis through suppression of active caspase-3 and Bax expression with restoration of Bcl-2 levels. The 20 mg/kg dose consistently produced greater biochemical, molecular, and histopathological protection than the lower dose. In conclusion, UPA confers significant protection against LPS/D-GalN-induced ALF through coordinated suppression of oxidative stress, inflammatory signaling, and apoptosis, primarily associated with inhibition of the IFN-γ/JAK1/STAT1 and TNF-α/NF-κB/MAPK pathways and modulation of the Bax/Bcl-2 ratio, underscoring its viability as a promising therapeutic candidate for ALF.

## 1. Introduction

Acute liver failure (ALF) is a life-threatening syndrome marked by abrupt onset of severe hepatocellular injury, abrupt deterioration of hepatic synthetic function, and high short-term mortality in individuals without preexisting advanced liver disease [1]. Despite progress in supportive intensive care, prognosis remains poor in severe cases, and liver transplantation is still the only definitive intervention for many patients [2]. This has driven continued interest in identifying pharmacological agents capable of interrupting the molecular pathways responsible for fulminant hepatocellular destruction and thereby limiting progression to irreversible liver failure [3].

Among experimental systems used to study ALF, the lipopolysaccharide and D-galactosamine (LPS/D-GalN) model is one of the best established because it reproduces many of the biochemical, inflammatory, and histopathological characteristics of human ALF [4]. D-GalN selectively depletes uridine nucleotides in hepatocytes, impairs macromolecular synthesis, and renders the liver highly susceptible to endotoxin-mediated injury [5]. LPS provokes an exaggerated innate immune response with marked cytokine release and rapid hepatocellular death [6]. The model is therefore particularly suitable for investigating inflammation-driven and apoptosis-dominated hepatic injury [7].

Evidence from cytokine-driven cell injury models indicates that tumor necrosis factor (TNF-ɑ) can synergize with interferon (INF-γ)-related signaling to induce Janus kinase (JAK1) and signal transducer and activator of transcription (STAT1)-dependent cytotoxic cascade, with sustained STAT1 activation serving as an important driver of downstream tissue injury [8]. Once activated, this pathway can converge on mitogen-activated protein kinase (MAPK) signaling, including c-Jun N-terminal kinase (JNK), p38 MAPK, and extracellular signal regulated kinase (ERK), which further intensify inflammatory gene transcription and stress responses. Downstream activation of nuclear factor kappa B (NF-κB) then enhances the expression of TNF-ɑ, inducible nitric oxide synthase (iNOS), and other inflammatory mediators, creating a self-amplifying inflammatory loop [9].

Upadacitinib (UPA) is an oral selective JAK1 inhibitor that has been approved for several immune-mediated inflammatory disorders, including rheumatoid arthritis, psoriatic arthritis, axial spondyloarthritis, atopic dermatitis, ulcerative colitis, and Crohn’s disease [10]. A recent report has indicated the potential of UPA to attenuate the injury kinase signaling and apoptotic execution activated in hepatic steatosis [11]. Nevertheless, the potential beneficial effect of UPA in LPS/D-GalN-induced ALF has not been studied yet. Consequently, the current study was undertaken to investigate whether UPA can blunt the oxidative/nitrosative, inflammatory, and apoptotic network that is activated in LPS/D-GalN-sensitized liver injury.

## 2. Materials and Methods

### 2.1. Drugs and Chemicals

LPS; from *Escherichia coli* serotype O111:B4 and D-GalN were procured from Sigma-Aldrich Chemical Co. (Saint Louis, MO, USA). The LPS was reconstituted in sterile saline to yield a stock solution of 1 mg/mL, which was then aliquoted, stored at −20 °C, and freshly diluted immediately prior to administration. D-GalN was dissolved in sterile 0.9% saline to a final concentration of 70 mg/mL, assisted by gentle warming (not exceeding 37 °C) to ensure complete dissolution. UPA (Rinvoq^®^) was obtained from AbbVie Ltd. (Maidenhead, UK). All other chemicals and reagents used in this study were of the highest grade available.

### 2.2. Kits and Antibodies

All details about kits and antibodies used in the current study are provided in Supplementary Tables S1 and S2, respectively.

### 2.3. Experimental Animals

Adult male BALB/c mice (weighing approximately 25 g) were sourced from VACSERA (Giza, Egypt). Animals were maintained in standard plastic cages under a controlled environment with a constant temperature of 25 °C and a 12 h light/dark cycle. Throughout the study, the mice had ad libitum access to water and a standard pellet diet. All experimental procedures were evaluated and approved by the Mansoura University Animal Care and Use Committee (approval code: MU-ACUC [PHARM.MS.24.09.108]).

### 2.4. Experimental Design

Thirty mice were randomly divided into five groups (*n* = 6 per group). The first group served as the normal control (negative CTRL) group and received the vehicles only [distilled water orally along with sterile saline intraperitoneally (i.p.)] for four consecutive days. The second group served as the drug CTRL (UPA CTRL) group and received UPA orally at a dose of 20 mg/kg without induction of hepatic injury. The third group represented the positive CTRL group and received distilled water orally for four consecutive days and i.p. injected with LPS (10 µg/kg) and D-GalN (700 mg/kg) once on the 4th day to induce ALF. The fourth and fifth groups were administered orally with UPA (10 and 20 mg/kg, respectively) once daily for four successive days and intoxicated with LPS/D-GalN on the 4th day. The dose selection of LPS/D-GalN was guided by a previous study by Wang [12] and the UPA doses were based on Anbar and Rotta [13,14].

### 2.5. Sample Collection and Preparation

Eight hours following the administration of LPS/D-GalN, mice were anesthetized via an i.p injection of thiopental sodium (70 mg/kg) [15]. Blood was drawn via cardiac puncture and subjected to centrifugation at 1000× *g* for 10 min at 4 °C; the isolated serum was subsequently utilized for biochemical assays. Following euthanasia, the liver was promptly excised and rinsed with ice-cold saline. The right median lobe was separated and fixed in 10% (*v*/*v*) neutral-buffered formalin to prepare paraffin blocks for histopathological and immunohistochemical evaluations. The left median lobe was dissected and divided into two sections: one section was stored at −80 °C for Western blot analysis, while the other was homogenized in phosphate-buffered saline (10% *w*/*v*) and centrifuged at 3000× *g* for 20 min at 4 °C [16]. The resulting supernatant was harvested and preserved at −80 °C for downstream biochemical and ELISA assessments.

### 2.6. Assessment of Hepatic Function

The serum concentrations of hepatic enzymes, alanine aminotransferase (ALT), aspartate aminotransferase (AST), alkaline phosphatase (ALP), and gamma glutamyl transferase (γ-GT), were measured following standardized biochemical techniques using commercially available assay kits (Supplementary Table S1).

### 2.7. Assessment of Hepatic Redox Status

Oxidative stress markers and antioxidant defense profiles were evaluated within the liver tissue homogenates. Lipid peroxidation was determined by quantifying malondialdehyde (MDA) levels. Antioxidant status was assessed by determining the levels of total antioxidant capacity (TAC). In addition, the total nitrite/nitrate (NO) was assessed. All oxidative stress and antioxidant indicators were measured using commercially available assay kits (Supplementary Table S1), following the manufacturer’s instructions.

### 2.8. Histopathological Assessment of the Liver

Paraffin-embedded blocks were sectioned at a thickness of 5 μm, mounted onto glass slides, and subjected to hematoxylin and eosin (H&E) staining for histopathological evaluation. All H&E-stained slides were digitally imaged using a 3DHISTECH Pannoramic Scan II whole slide scanner (3DHISTECH, Budapest, Hungary) equipped with a 20× objective lens at a resolution of 0.5 μm/pixel, generating high-resolution whole slide images (WSIs). The scanned slides were initially reviewed using CaseViewer 2.2.1 software (3DHISTECH, Budapest, Hungary) for quality assessment prior to computational analysis.

Digital image analysis was subsequently performed using QuPath software (v0.6.0), an open-source digital pathology platform [17]. Whole slide images were imported into QuPath and image metadata, including scanner type, objective magnification, and pixel size, were recorded. Image type was configured with default Brightfield settings for H&E-stained sections. Stain vectors were estimated separately for each batch before analysis. For histopathological evaluation, the entire hepatic parenchyma was annotated while excluding large blood vessels and tissue artifacts to ensure uniform tissue assessment.

Neutrophil identification was performed using QuPath’s Train Object Classifier based on a random trees algorithm trained through manual annotation of representative neutrophil and non-neutrophil populations. For neutrophil quantification, ten high power fields containing the highest aggregates of polymorphonuclear leukocytes with segmented nuclei were selected, annotated, and quantified for each experimental group, and the average number of infiltrating neutrophils was subsequently calculated [18]. Histopathological injury was further investigated where the percentage of areas showing inflammatory and necrotic lesions within annotated regions was quantified.

### 2.9. Immunohistochemical (IHC) Assessment of the Liver

IHC analysis was conducted to evaluate the hepatic expression levels of NF-κB p65, iNOS, and active caspase-3. Following deparaffinization and rehydration, the embedded liver sections were processed via standard IHC protocols prior to incubation with the respective primary antibodies. All immunohistochemically stained slides were digitally scanned using a 3DHISTECH Pannoramic Scan II whole slide scanner (3DHISTECH, Budapest, Hungary) equipped with a 20× objective lens at a resolution of 0.5 μm/pixel, generating high-resolution whole slide images (WSIs). The scanned slides were initially reviewed using CaseViewer software (3DHISTECH, Budapest, Hungary) for quality assessment prior to computational analysis.

Immunohistochemically stained sections were visualized and photographed using QuPath software (v0.6.0). Image type was set to Brightfield (H-DAB) and stain vectors were re-estimated using representative positive and negative controls. Positive cell detection was performed to classify cells as positive or negative based on DAB optical density (OD) thresholds and to segment positive staining (membranous, cytoplasmic, and nuclear as appropriate for each marker). Cell detection was integrated with staining classification to assign positivity at the single-cell level. The total percentage of positive cells was calculated automatically.

### 2.10. Determination of Inflammatory Cytokines and Apoptotic Markers

Using specific corresponding enzyme-linked immunosorbent assay (ELISA) kits (Supplementary Table S1), inflammatory cytokine (TNF-ɑ, INF-γ, p-JAK1, p-STAT1, JNK, and p38) and apoptotic markers [pro-apoptotic Bcl-2 Associated X-protein (Bax) and anti-apoptotic B-cell lymphoma 2 (Bcl-2)], protein expression levels were determined in liver homogenates relative to total protein content of the samples assessed by the Bradford method and according to standardized laboratory protocols.

### 2.11. Western Blot Analysis

Protein expression of phosphorylated extracellular signal-regulated kinases (p-ERK) was determined using Western blot technique. Briefly, liver tissue homogenates were prepared and equal quantities of protein samples were resolved via sodium dodecyl sulfate-polyacrylamide gel electrophoresis (SDS-PAGE). Following electrophoretic resolution, the separated proteins were electro-transferred onto membrane surfaces and incubated with primary antibodies directed against p-ERK1/2 (Supplementary Table S2). Following incubation with secondary antibodies, chemiluminescent detection was applied to visualize protein bands and quantification performed via image analysis software. Expression levels of target proteins were normalized relative to an internal loading control protein, β-actin.

### 2.12. Statistical Analysis

Statistical analyses were performed utilizing GraphPad Prism software (version 9.0.1; GraphPad Software Inc., San Diego, CA, USA). Data are expressed as the mean ± standard deviation (SD). Differences among the experimental groups were evaluated using a one-way analysis of variance (ANOVA), followed by the Tukey–Kramer post hoc test for multiple comparisons. Statistical significance was defined as a p-value of less than 0.05.

## 3. Results

### 3.1. UPA Mitigates Liver Dysfunction in ALF

**Figure 1:**
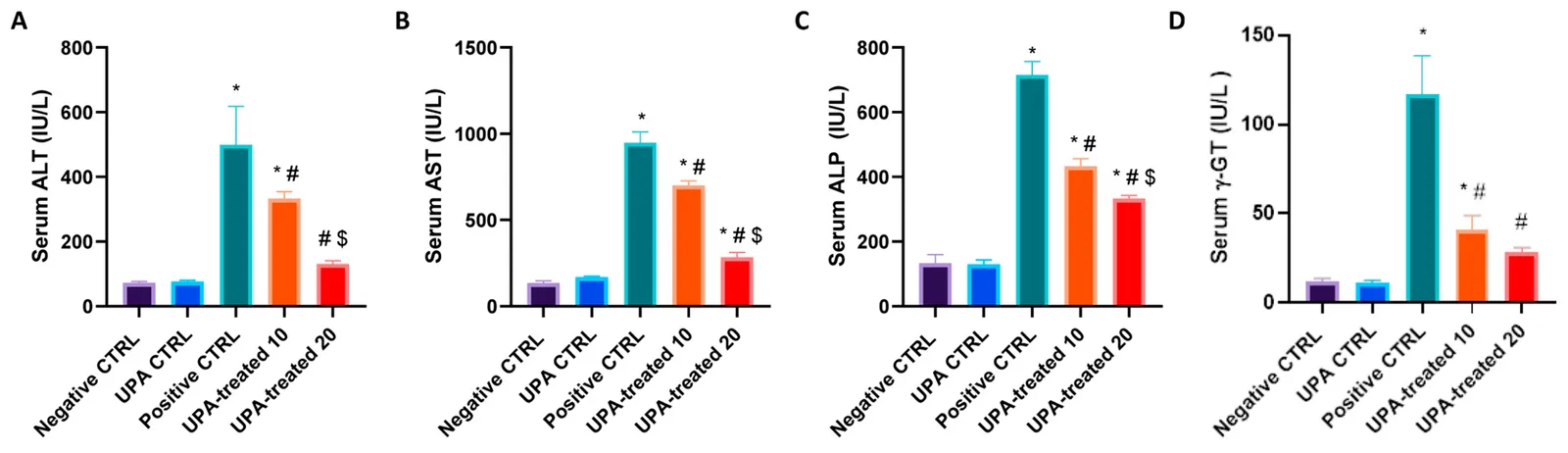
Effect of upadacitinib (UPA) pretreatment on serum biochemical markers of hepatic injury in lipopolysaccharide/D-galactosamine-intoxicated mice. (**A**) Alanine aminotransferase (ALT), (**B**) aspartate aminotransferase (AST), (**C**) alkaline phosphatase (ALP), and (**D**) gamma glutamyl transferase (γ-GT) serum activities. Data are expressed as mean ± SD (n = 6). *, #, $ significant difference compared to the negative control (CTRL), positive CTRL, UPA-treated 10 groups, respectively.

Intoxication with LPS/D-GalN yielded a marked hepatocellular injury and liver dysfunction, as evidenced by marked elevations in serum ALT, AST, ALP, and γ-GT levels by about 6.6-, 7-, 5.7-, and 9.7-fold, corresponding to increases of 557.6%, 601.8%, 472.0%, and 873.1%, respectively, relative to the negative CTRL group (Figure 1A–D, respectively). Pretreatment with UPA at 10 and 20 mg/kg significantly alleviated the LPS/D-GalN-induced hepatic injury, as demonstrated by reductions in ALT by 29.8% and 71.8%, AST by 25.1% and 71.3%, ALP by 39.3% and 46.6%, and GGT by 64.1% and 74.9%, respectively, relative to the positive CTRL group. Overall, the higher dose conferred a more pronounced hepatoprotective effect, particularly for ALT, AST, and ALP, while the UPA CTRL group showed no significant difference from the negative CTRL one.

### 3.2. UPA Restores Hepatic Redox Homeostasis in ALF

**Figure 2:**
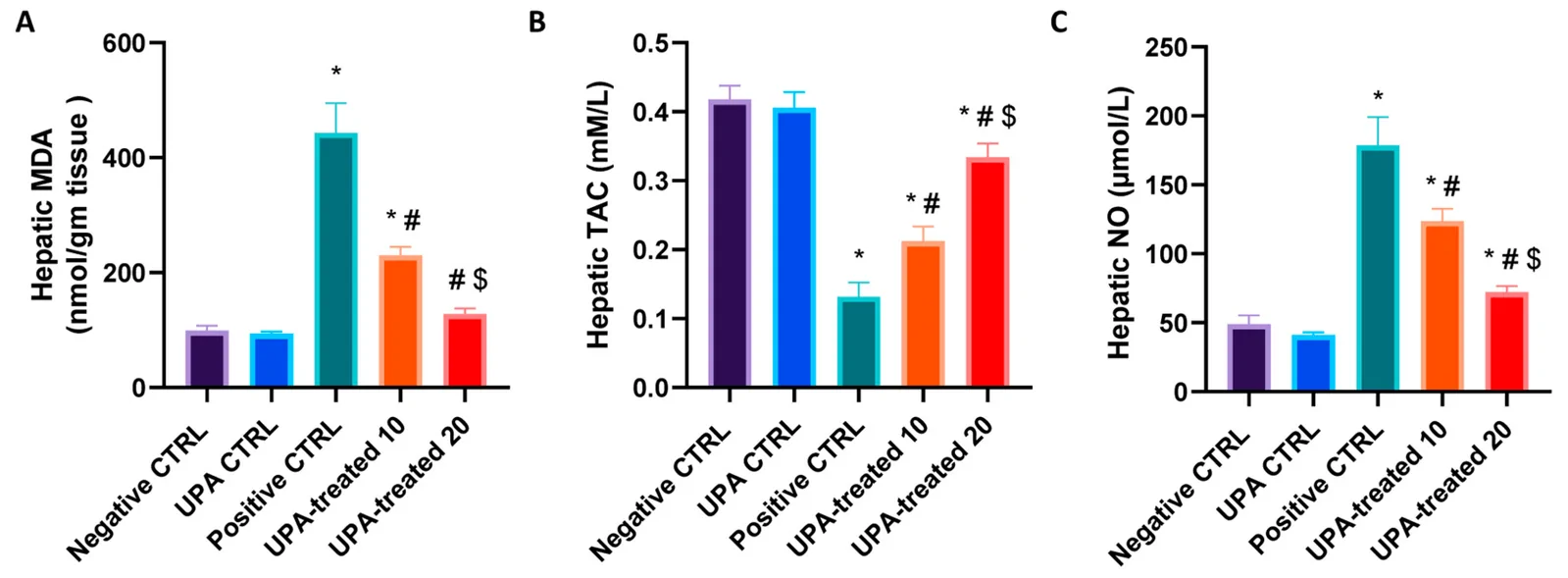
Effect of upadacitinib (UPA) pretreatment on hepatic oxidative and nitrosative stress markers in lipopolysaccharide/D-galactosamine-intoxicated mice. (**A**) Hepatic malondialdehyde (MDA), (**B**) hepatic total antioxidant capacity (TAC), and (**C**) hepatic total nitrite/nitrate (NO) levels. Data are expressed as mean ± SD (n = 6). *, #, $ significant difference compared to the negative control (CTRL), positive CTRL, UPA-treated 10 groups, respectively.

LPS/D-GalN administration markedly disturbed hepatic redox balance, as shown by the about 4.6-fold rise in hepatic MDA, equivalent to a 364.4% increase, a 67.0% depletion in hepatic TAC, reaching 0.33-fold of the negative CTRL value, and a 3.8-fold increase in hepatic NO, equivalent to 278.4%, compared with the negative CTRL group (Figure 2A–C, respectively). Pretreatment with UPA at 10 and 20 mg/kg significantly ameliorated these alterations, reducing MDA by 48.3% and 71.1%, restoring TAC by 48.5% and 143.6%, and lowering NO by 31.7% and 60.5%, respectively, relative to the positive CTRL group. The higher dose showed a clearly stronger antioxidant and anti-nitrosative effect, while the UPA CTRL group remained comparable to the negative CTRL group.

### 3.3. UPA Ameliorates Histopathological Liver Injury in ALF

**Figure 3:**
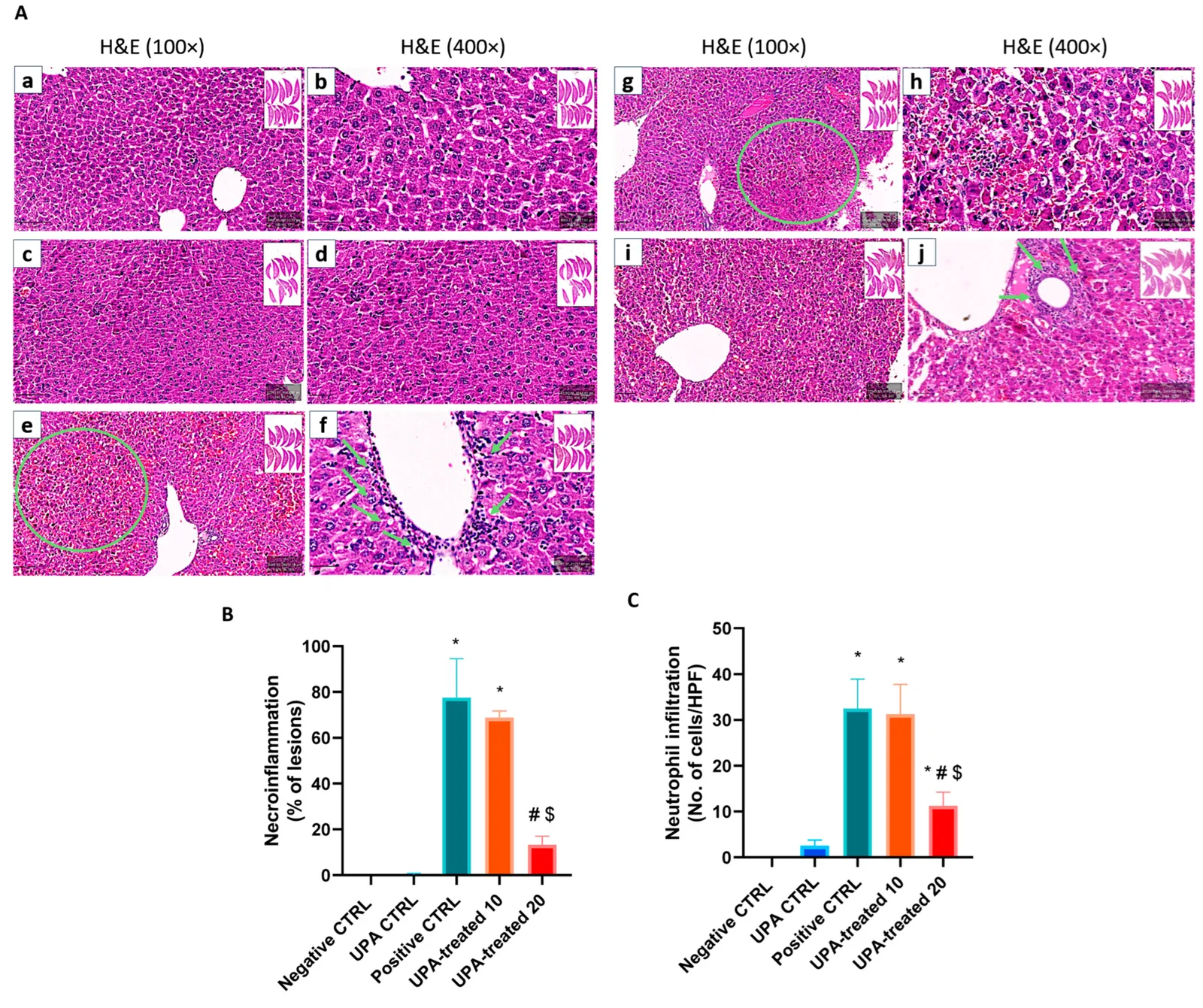
Effect of upadacitinib (UPA) pretreatment on hepatic histopathological alterations in lipopolysaccharide/D-galactosamine-intoxicated mice. (**A**) Representative haematoxylin and eosin (H&E)-stained liver sections from the negative control (CTRL) group (**a**,**b**), UPA CTRL group (**c**,**d**), positive CTRL group (**e**,**f**), UPA-treated 10 (**g**,**h**), and UPA-treated 20 (**i**,**j**). Lower magnification (×100); higher magnification ×400. (**B**) Corresponding necroinflammation percentage. (**C**) Neutrophil infiltration count. The green arrows show neutrophil infiltration while the green circles show inflammation and necrosis. Data are expressed as mean ± SD. *, #, $ significant difference compared to the negative control (CTRL), positive CTRL, UPA-treated 10 groups, respectively.

In harmony with the biochemical findings, H&E-stained liver sections from the negative CTRL (Figure 3A(a,b)) and UPA CTRL (Figure 3A(c,d)) groups showed preserved hepatic architecture, intact hepatic cords, and essentially normal lobular organization, with no appreciable necroinflammatory damage. In contrast, the positive CTRL group (Figure 3A(e,f)) exhibited pronounced hepatic injury characterized by focal necroinflammatory lesions, hepatocellular degeneration, and dense inflammatory cell infiltration, particularly neutrophilic infiltration around vascular and parenchymal areas. Quantitatively, necroinflammation reached about 77.5% of lesions and average neutrophil count reached about 31 cells/10 HPFs in the positive CTRL group (Figure 3B and C, respectively). Pretreatment with UPA 10 mg/kg (Figure 3A(g,h)) afforded only limited, statistically nonsignificant histological improvement, lowering necroinflammation by 11.3%, while neutrophilic infiltration count remained essentially unchanged relative to the positive CTRL group. By contrast, UPA 20 mg/kg (Figure 3A(i,j)) significantly mitigated hepatic damage, reducing necroinflammation by 82.9% and neutrophilic infiltration count by 64.0%, with substantial preservation of hepatic architecture and only mild residual inflammatory changes.

### 3.4. UPA Suppresses Inflammatory Cytokine Production and JAK1/STAT1 Signaling in ALF

**Figure 4:**
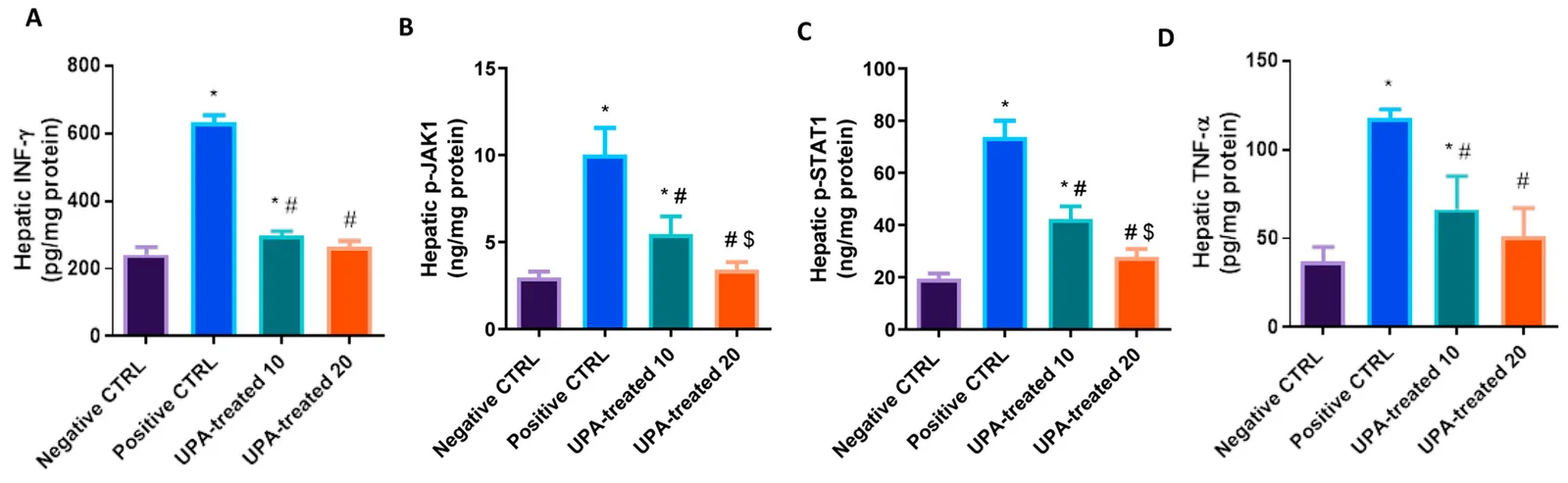
Effect of upadacitinib (UPA) pretreatment on hepatic inflammatory cytokines and JAK1/STAT1 signaling pathway in lipopolysaccharide/D-galactosamine-intoxicated mice. (**A**) Interferon gamma (IFN-γ), (**B**) phosphorylated Janus kinase 1 (p-JAK1), (**C**) phosphorylated signal transducer and activator of transcription 1 (p-STAT1), and (**D**) tumor necrosis factor alpha (TNF-α). Data are expressed as mean ± SD (n = 6). *, #, $ significant difference compared to the negative control (CTRL), positive CTRL, UPA-treated 10 groups, respectively.

Administration of LPS/D-GalN markedly activated inflammatory and cytokine-related signaling, as evidenced by elevations in hepatic IFN-γ, p-JAK1, p-STAT1, and TNF-α levels by about 2.6-, 3.9-, 3.80-, and 3.2-fold, corresponding to 161.1%, 238.1%, 279.7%, and 217.0%, respectively, compared with the negative CTRL group (Figure 4A–D, respectively). Pretreatment with UPA at 10 and 20 mg/kg significantly blunted these changes, reducing IFN-γ by 52.9% and 58.2%, p-JAK1 by 45.5% and 65.9%, p-STAT1 by 42.5% and 62.4%, and TNF-α by 43.6% and 56.6%, respectively, relative to the positive CTRL group. The ameliorative effect of the higher dose was particularly evident for p-JAK1 and p-STAT1, where it also exceeded the effect of the lower dose, indicating a dose-responsive inhibition of the JAK1/STAT1 axis.

### 3.5. UPA Downregulates iNOS and NF-κB p65 Immunoexpression in ALF

**Figure 5:**
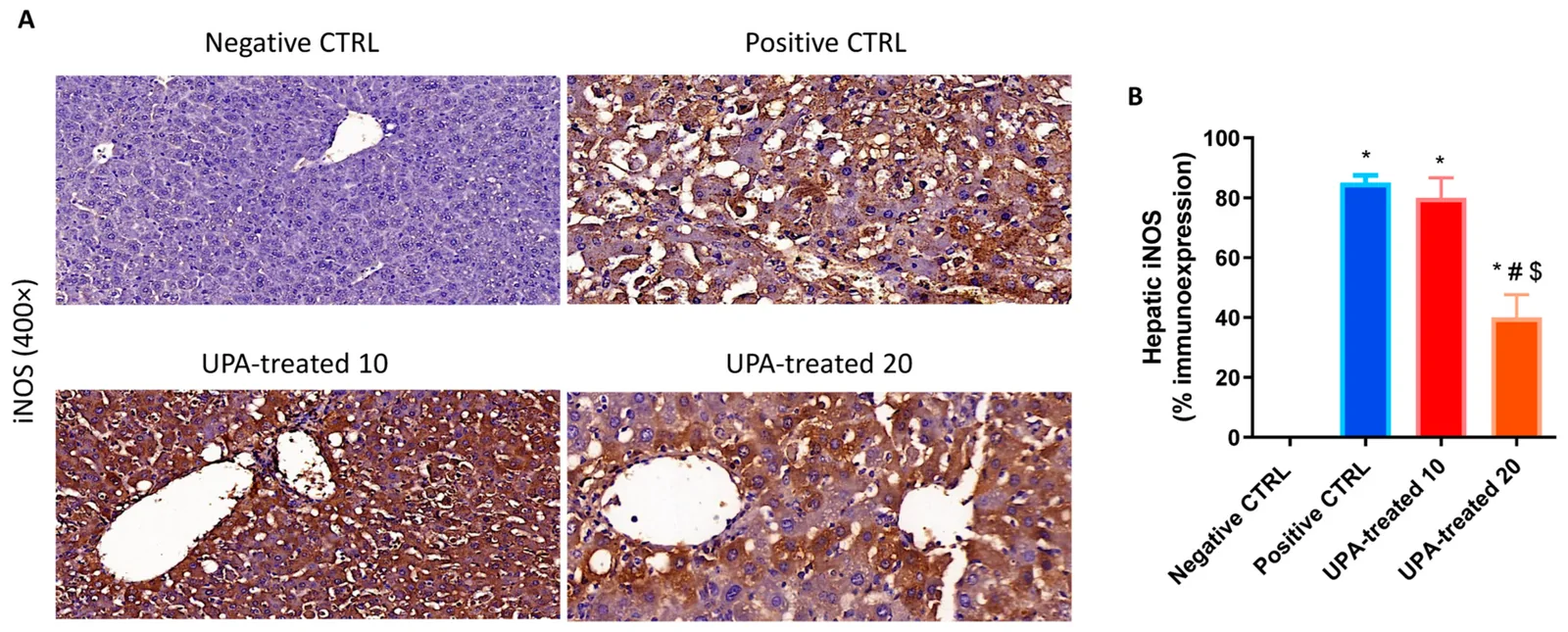
Effect of upadacitinib (UPA) pretreatment on hepatic inducible nitric oxide synthase (iNOS) immunoexpression in lipopolysaccharide/D-galactosamine-intoxicated mice. (**A**) Representative immunohistochemical images from the different experimental groups showing hepatic iNOS expression (magnification ×400). (**B**) Corresponding percentage of hepatic iNOS immunoexpression. Data are expressed as mean ± SD. *, #, $ significant difference compared to the negative control (CTRL), positive CTRL, UPA-treated 10 groups, respectively.

Immunohistochemical analysis revealed negligible iNOS immunoexpression in the negative CTRL liver sections, whereas the positive CTRL group displayed marked diffuse iNOS positivity (Figure 5A), reaching about 83.8% immunoexpression (Figure 5B). Pretreatment with UPA 10 mg/kg produced only a slight, statistically nonsignificant reduction to about 78.9%. In contrast, UPA 20 mg/kg significantly reduced hepatic iNOS immunoexpression to about 39.1%, representing a 53.4% decline, or approximately 2.15-fold attenuation, relative to the positive CTRL group.

**Figure 6:**
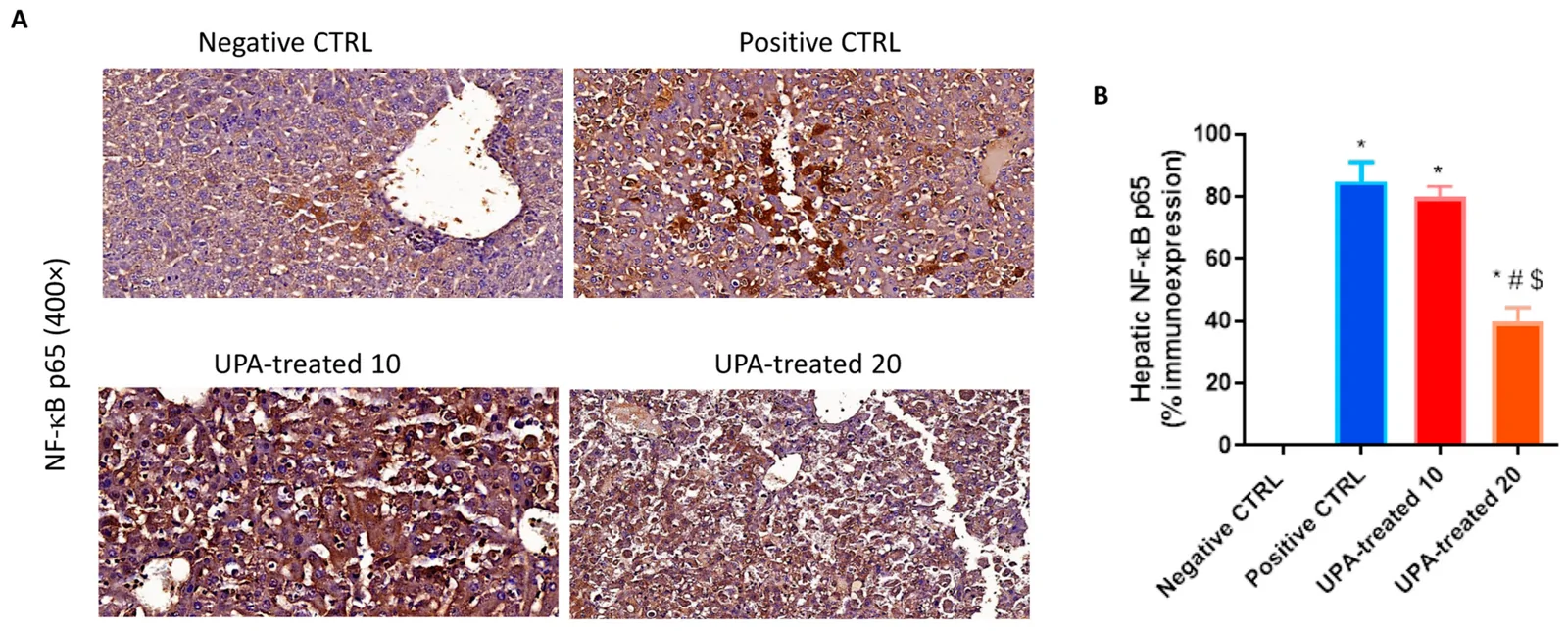
Effect of upadacitinib (UPA) pretreatment on hepatic nuclear factor kappa B (NF-κB p65) immunoexpression in lipopolysaccharide/D-galactosamine-intoxicated mice. (**A**) Representative immunohistochemical images from the different experimental groups showing hepatic NF-κB p65 expression (magnification ×400). (**B**) Corresponding percentage of hepatic NF-κB p65 immunoexpression. Data are expressed as mean ± SD. *, #, $ significant difference compared to the negative control (CTRL), positive CTRL, UPA-treated 10 groups, respectively.

A similar pattern was observed for hepatic NF-κB p65 immunoexpression. The negative CTRL group exhibited minimal cytoplasmic staining, whereas the positive CTRL group showed intense nuclear NF-κB p65 positivity (Figure 6A), reaching about 84% immunoexpression (Figure 6B). UPA 10 mg/kg caused only a minor, nonsignificant decrease to about 78.9%, while UPA 20 mg/kg significantly suppressed NF-κB p65 expression to about 38.9%, corresponding to a 53.4% reduction, or roughly 2.16-fold attenuation, compared with the positive CTRL group. These data indicate that the higher UPA dose exerted a clear inhibitory effect on the NF-κB/iNOS inflammatory arm.

### 3.6. UPA Attenuates MAPK Signaling Activation

**Figure 7:**
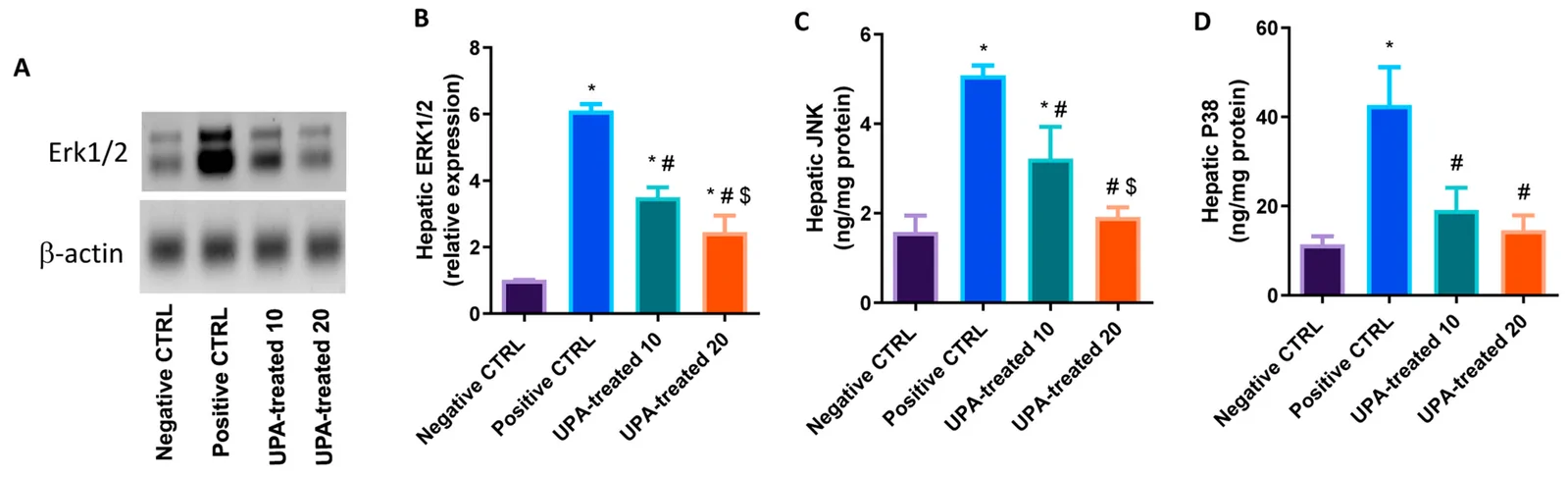
Effect of upadacitinib (UPA) pretreatment on hepatic mitogen-activated protein kinase (MAPK) signaling in lipopolysaccharide/D-galactosamine-intoxicated mice. (**A**) Representative Western blot images showing extracellular signal regulated kinase 1/2 (ERK1/2) protein expression and β-actin as loading control. (**B**) Relative hepatic ERK1/2 expression. (**C**) Hepatic c-Jun N-terminal kinase (JNK) levels. (**D**) Hepatic p38 MAPK levels. Data are expressed as mean ± SD (n = 6 for ELISA measurements and n = 3 for Western blot analysis). *, #, $ significant difference compared to the negative control (CTRL), positive CTRL, UPA-treated 10 groups, respectively.

LPS/D-GalN challenge also markedly activated MAPK-related signaling, as demonstrated by representative ERK1/2 immunoblots (Figure 7A) and densitometric analysis together with quantification of JNK and p38 levels (Figure 7B–D). Relative to the negative CTRL group, hepatic ERK1/2 expression increased by about 6.0-fold, equivalent to 502.0%, while JNK and p38 increased by 3.2- and 3.7-fold, corresponding to 220.5% and 273.7%, respectively. Pretreatment with UPA at 10 and 20 mg/kg significantly attenuated these elevations, reducing ERK1/2 by 42.6% and 59.8%, JNK by 36.6% and 62.2%, and p38 by 55.1% and 65.8%, respectively, compared with the positive CTRL group. The higher dose produced a stronger inhibitory effect on ERK1/2 and JNK, while both doses also markedly suppressed p38 activation.

### 3.7. UPA Alleviates Hepatocellular Apoptosis

**Figure 8:**
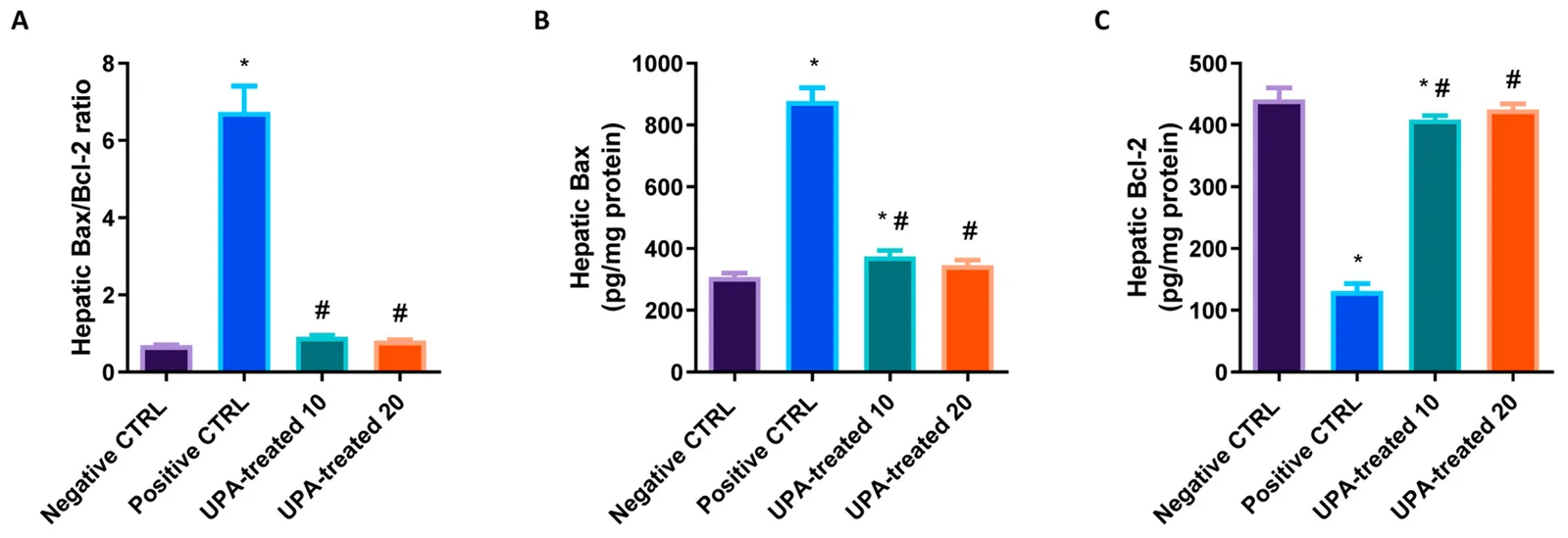
Effect of upadacitinib (UPA) pretreatment on hepatic apoptotic Bcl-2 associated X-protein (Bax)/B-cell lymphoma 2 (Bcl-2) signaling in lipopolysaccharide/D-galactosamine-intoxicated mice. (**A**) Hepatic Bax/Bcl-2 ratio. (**B**) Hepatic Bax levels. (**C**) Hepatic Bcl-2 levels. Data are expressed as mean ± SD. *, # significant difference compared to the negative control (CTRL) and positive CTRL groups, respectively.

The LPS/D-GalN challenge shifted the Bax/Bcl-2 balance toward apoptosis (Figure 8A), as hepatic Bax rose by about 2.9-fold, equivalent to 184.5%, while hepatic Bcl-2 declined by 70.3%, reaching only 0.30-fold of the negative CTRL value, compared with the negative CTRL group (Figure 8B and C, respectively). Pretreatment with UPA 10 and 20 mg/kg significantly counteracted this imbalance, decreasing Bax by 57.2% and 60.5%, respectively, and increasing Bcl-2 by 212.2% and 224.4%, respectively, relative to the positive CTRL group. Collectively, these results demonstrate that UPA restored the Bax/Bcl-2 profile toward cell survival, with the higher dose producing a numerically greater anti-apoptotic effect.

**Figure 9:**
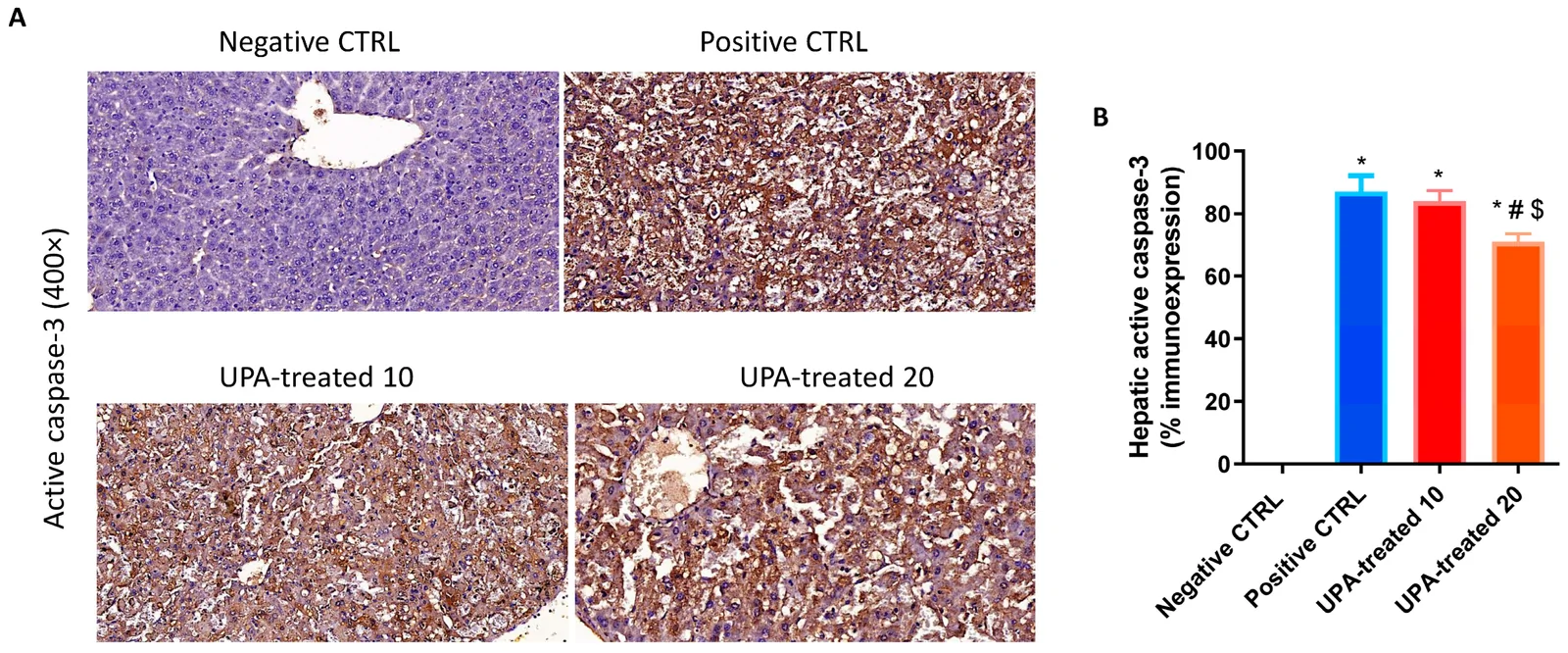
Effect of upadacitinib (UPA) pretreatment on hepatic active caspase-3 immunoexpression in lipopolysaccharide/D-galactosamine-intoxicated mice. (**A**) Representative immunohistochemical images from the different experimental groups showing active caspase-3 expression in hepatic tissue (magnification ×400). (**B**) Corresponding percentage of hepatic active caspase-3 immunoexpression. Data are expressed as mean ± SD. *, #, $ significant difference compared to the negative control (CTRL), positive CTRL, UPA-treated 10 groups, respectively.

Consistent with these findings, immunohistochemical staining for active caspase-3 showed negligible expression in the negative CTRL group, whereas the positive CTRL group exhibited intense positive staining (Figure 9A) involving about 85.5% of hepatic cells (Figure 9B). UPA 10 mg/kg induced only a mild, statistically nonsignificant decline to about 82.6%. By contrast, UPA 20 mg/kg significantly reduced active caspase-3 immunoexpression to about 69.7%, corresponding to about 1.2-fold attenuation, versus the positive CTRL group, indicating a partial but significant suppression of apoptotic execution.

## 4. Discussion

The poor prognosis of ALF and the limited therapeutic options available highlight the critical necessity for targeted therapeutic interventions capable of attenuating early hepatocellular injury and interrupting progression toward irreversible fulminant hepatic failure [2,3]. In this context, the LPS/D-GalN model remains one of the most clinically relevant experimental models because it closely reproduces the necroinflammatory, oxidative, and apoptotic features observed in ALF [4].

The present study demonstrates that UPA, especially at the higher dose, effectively mitigates LPS/D-GalN-induced ALF through coordinated antioxidant, anti-inflammatory, and anti-apoptotic actions. Collectively, these beneficial effects were associated with modulation of inflammatory and oxidative stress signaling pathways, particularly the IFN-γ/JAK1/STAT1 cascade, resulting in suppression of proinflammatory mediators, attenuation of hepatocellular apoptosis, and reduction in oxidative tissue injury.

UPA significantly attenuated LPS/D-GalN-induced elevations in serum ALT, AST, ALP, and γ-GT levels, which represent established biomarkers of hepatocellular injury and hepatic enzyme leakage [19]. These biochemical findings suggest substantial preservation of hepatic integrity following UPA administration.

Oxidative stress represents a major starting event in LPS/D-GalN-induced hepatotoxicity and results from disruption of the balance between oxidant generation and antioxidant defense systems, thereby promoting inflammatory progression and activation of apoptotic signaling pathways [20]. In the present study, UPA effectively interrupted this early oxidative phase and restored redox homeostasis, as evidenced by reduced MDA levels, increased TAC, and diminished NO production. Previous evidence suggests that UPA exerts indirect antioxidant effects through suppression of upstream inflammatory signaling pathways involved in reactive oxygen species (ROS) generation [11].

Histopathological findings further confirmed the protective effects of UPA. Herein, LPS/D-GalN induced extensive hepatocellular degeneration, necrosis, and prominent neutrophil infiltration as previously reported by Chen [21], whereas UPA pretreatment preserved hepatic architecture, reduced necroinflammatory changes, and markedly limited inflammatory cell accumulation. Notably, UPA at 20 mg/kg produced substantially greater suppression of necrosis and inflammatory infiltration than the lower dose, highlighting the dose-responsive nature of its hepatoprotective actions and supporting a major anti-inflammatory role for UPA in limiting progression of liver injury.

The findings of the current study showed that UPA significantly reduced hepatic IFN-γ expression and suppressed phosphorylation of JAK1 and STAT1 in the liver. Moreover, UPA markedly repressed hepatic TNF-α level and iNOS expression, particularly at the higher dose, supporting inhibition of JAK1/STAT1-driven inflammatory signaling as a major protective mechanism. IFN-mediated signaling represents an important upstream pathway involved in inflammatory amplification during hepatic injury. IFN-γ activates the JAK1/STAT1 axis and results in phosphorylation of STAT1, which subsequently translocates into the nucleus and promotes transcription of several inflammatory mediators, including iNOS and TNF-α, thereby promoting oxidative stress and tissue injury [22,23]. TNF-α further amplifies inflammatory signaling through reciprocal interactions with JAK/STAT and NF-κB pathways, creating a self-perpetuating inflammatory cycle [24]. Experimental studies have demonstrated that suppression of JAK1 signaling attenuates STAT1 activation and downstream inflammatory mediator production [13,24].

Our findings further revealed that the higher UPA dose markedly suppressed hepatic NF-κB expression, indicating that inhibition of NF-κB-dependent inflammatory signaling contributes substantially to UPA-mediated hepatoprotection. NF-κB signaling occupies a central role in orchestrating inflammatory responses during LPS/D-GalN-induced liver injury. Activation of NF-κB promotes the transcription of multiple inflammatory mediators, including TNF-α, thereby sustaining cytokine release and inflammatory amplification [25,26].

The present results demonstrated that UPA significantly reduced the activation of ERK1/2, JNK, and p38, indicating that inhibition of MAPK signaling contributes to its hepatoprotective and anti-inflammatory effects. MAPK signaling plays a pivotal role in LPS/D-GalN-induced ALF and functions as a major downstream mediator of inflammatory and apoptotic responses. Activation of ERK, JNK, and p38 pathways promotes inflammatory transcription, oxidative stress, TNF-α-mediated injury, neutrophil recruitment, and hepatocyte apoptosis. Moreover, MAPK signaling interacts with cytokine and NF-κB pathways, thereby amplifying hepatic inflammation and tissue injury [4,7]. Consistent with our results, previous studies suggest that selective JAK inhibition attenuates inflammatory kinase signaling and downstream MAPK activation [11,24].

The anti-apoptotic effect of UPA was further illustrated in the present study by a significant decrease in Bax while increasing in Bcl-2 hepatic expression, alongside reduced caspase-3 activation especially at the higher dose. These data imply that UPA limits hepatocyte apoptosis through suppression of upstream inflammatory triggers. Extensive hepatocyte apoptosis is a central mechanism underlying LPS/D-GalN-induced ALF and is characterized by increased expression of the pro-apoptotic Bax, decreased expression of the anti-apoptotic Bcl-2, and caspase-3 activation [27].

Comprehensively, this study suggests a unified mechanistic model in which suppressing the JAK1/STAT1 signaling represents a key upstream event responsible for inhibition of multiple downstream pathogenic processes. As previously reported, activation of JAK1/STAT1 signaling stimulates the transcription of different pro-inflammatory cytokines. Furthermore, activated STAT1 interacts with both the NF-κB and MAPK axes, which boosts inflammatory responses. Crosstalk between these signaling cascades induces the phosphorylation of ERK1/2, JNK, and p38 MAPKs. These events collectively trigger the expression of pro-inflammatory mediators, such as TNF-α and iNOS, resulting in excessive production of ROS and redox imbalance. Persistent redox imbalance subsequently disrupts mitochondrial integrity by stimulating the intrinsic apoptotic pathway via upregulating Bax, downregulating Bcl-2, and activating caspase-3 [22,28]. Accordingly, inhibiting IFN-γ/JAK1/STAT1 signaling may simultaneously repress NF-κB- and MAPK-related inflammatory signaling, suppress oxidative stress, and limit hepatocyte apoptosis.

## 5. Conclusions

This study demonstrates that UPA provides significant protection against ALF induced by LPS/D-GalN in mice by attenuating hepatocellular injury, inflammation, and cell death. UPA pretreatment reduced serum liver enzymes, preserved hepatic histoarchitecture, and limited necroinflammation and neutrophil infiltration, confirming a proven hepatoprotective effect. These benefits were mainly confined to the 20 mg/kg dose, which achieved greater biochemical, molecular, and histological protection than the 10 mg/kg dose. Mechanistically, UPA intervention was associated with suppressed IFN-γ/JAK1/STAT1 and TNF-α/NF-κB/MAPK-driven inflammatory signaling. In parallel, UPA pretreatment was accompanied by a decreased Bax/Bcl-2 ratio and attenuated caspase-3-dependent apoptosis. Overall, UPA emerges as a promising candidate capable of interrupting the oxidative, inflammatory, and cell death cascades central to ALF.

## 6. Future Directions and Limitations

Although our results illustrate coordinated inhibition of the IFN-γ/JAK1/STAT1, NF-κB, and MAPK signaling pathways upon UPA intervention, they do not set up a definitive causal hierarchy among these pathways. Therefore, the observed changes should be interpreted as associative. Future studies using pathway-specific inhibitors or genetic approaches are needed to further confirm the mechanistic interactions underlying the beneficial effect of UPA. While the current study shows the protective effect of UPA against ALF, future investigations should explore its potential therapeutic effect after ALF induction, as ALF cases typically present well after the onset of hepatic injury. Moreover, it will be favorable to include both male and female mice in future studies to fully validate the translational relevance of the observations.

## Data Availability

The original contributions presented in this study are included in the article/Supplementary Materials. Further inquiries can be directed to the corresponding author.

## Supplementary Materials

The following supporting information can be downloaded at: https://www.mdpi.com/article/10.3390/jox16040140/s1, Table S1: Assay kits; Table S2: Antibodies; File S1: Original images of Figure 3, Figure 5, Figure 6, Figure 7 and Figure 9.
