# Supplementary material for: The JAK1 Inhibitor Upadacitinib Curbs Acute Liver Failure via Suppressing IFN-γ/JAK1/STAT1 and TNF-α/NF-κB/MAPK Pathways and Modulating Bax/Bcl-2 Ratio
Source: J Xenobiot. 2026 Jul 29;16(4):140. doi: 10.3390/jox16040140 (PMC13514836; doi:10.3390/jox16040140)
Supplement: Supplementary file 1 [file jox-16-00140-s001.zip › jox-4390507-supplementary.pdf]

Supplementary Materials: The JAK1 Inhibitor Upadacitinib Curbs Acute Liver Failure via Suppressing IFN-γ/JAK1/STAT1 and TNF-α/NF-κB/MAPK Pathways and Modulating Bax/Bcl-2 Ratio

Abdulaziz F. Alhussaini, Sara H. Hazem, Eman A. Saad and Mahmoud Elshal

Table S1. Assay kits.

Kit	Supplier	Catalog No.
Mouse interferon γ (INF-γ) ELISA Kit	HCUSABIO	CSB-E04578m
Tyrosine-Protein Kinase JAK1 (JAK1) ELISA Kit for Mouse	MyBioSource	MBS2887172
Mouse STAT1(Signal Transducer And Activator Of Transcription 1) ELISA Kit	MyBioSource	MBS8819718
Mouse JNK(c-Jun N-terminal kinases) ELISA Kit	ELK Biotechnology	ELK0946
Mouse MAPK P38 ELISA Kit	AssayGenie	MOFI00965
Mouse TNF α ELISA Kit	MyBioSource	MBS825075
Bax ELISA Kit	LSBio(LifeSpan BioSci-ences,Inc.)	LS-F8604
Mouse Bcl-2 (Β-cell Lymphoma 2)ELISA Kit	MyBioSource	MBS2512543
Bradford Protein Assay Kit	BIO BASIC INC.	SK3041
Liquichek for ALT Kit	Agappe Diagnostic Ltd., In-dia SGOT	11409005
Liquichek for ALP Kit	Agappe Diagnostic Ltd., In-dia SGOT	11401008
Liquichek for AST Kit	Agappe Diagnostic Ltd., In-dia SGOT	11408005
Liquichek for GGT Kit	Agappe Diagnostic Ltd., In-dia SGOT	11416005
Lipid Peroxide (Malondialdehyde MDA) Kit	Bio-diagnostic, Egypt	MD2529
Nitric oxide Assay (NO) Kit	Bio-diagnostic, Egypt	NO2533
Total antioxidant capacity (TAC) kit	Bio-diagnostic, Egypt	TA2513

Table S2. Antibodies.

Antibody	Host	Clonality	Supplier	Catalog No.
β-actin Monoclonal Antibody	Mouse	Monoclonal	Thermo Fisher Scientific, USA	MA1-140
ERK1/2 Monoclonal Anti-body	Mouse	Monoclona	Thermo Fisher Scientific, USA	14-9108-82
